# Parasites in the changing world – Ten timely examples from the Nordic-Baltic region^☆^

**DOI:** 10.1016/j.parepi.2020.e00150

**Published:** 2020-05-05

**Authors:** Gunita Deksne, Rebecca K. Davidson, Kurt Buchmann, Age Kärssin, Muza Kirjušina, Inese Gavarāne, Andrea L. Miller, Guðný Rut Pálsdóttir, Lucy J. Robertson, Torill Mørk, Antti Oksanen, Vaidas Palinauskas, Pikka Jokelainen

**Affiliations:** a Institute of Food safety, Animal health and Environment “BIOR”, Lejupes Str. 3, Riga LV-1076, Latvia; b Faculty of Biology, University of Latvia, Jelgavas Str. 1, Riga LV-1004, Latvia; c Norwegian Veterinary Institute, Stakkevollvegen 23b, 9010 Tromsø, Norway; d Laboratory of Aquatic Pathobiology, Department of Veterinary and Animal Sciences, University of Copenhagen, Stigbøjlen 7, DK-1870 Frederiksberg C, Denmark; e Veterinary and Food Laboratory, Kreutzwaldi 30, 51006 Tartu, Estonia; f Institute of Veterinary Medicine and Animal Sciences, Estonian University of Life Sciences, Kreutzwaldi 62, 51006 Tartu, Estonia; g Institute of Life Sciences and Technology, Daugavpils University, Parādes Str. 1A, Daugavpils LV-5401, Latvia; h Norwegian Institute for Nature Research, Department for Terrestrial Ecology, Postboks 5685 Sluppen, 7485 Trondheim, Norway; i Institute for Experimental Pathology at Keldur, University of Iceland, Keldnavegur 3, IS-112 Reykjavík, Iceland; j Norwegian University of Life Sciences, Department of Food Safety and Infection Biology, Section for Microbiology, Immunology, and Parasitology, Parasitology Lab, Adamstuen Campus, Ullevålsveien 72, 0454 Oslo, Norway; k Finnish Food Authority (FINPAR), Elektroniikkatie 3, 90590 Oulu, Finland; l Nature Research Centre, Akademijos g. 2, LT-08412 Vilnius, Lithuania; m Laboratory of Parasitology, Department of Bacteria, Parasites & Fungi, Infectious Disease Preparedness, Statens Serum Institut, Artillerivej 5, 2300 Copenhagen S, Denmark

**Keywords:** Europe, Parasite, Epidemiology, Host, Climate change

## Abstract

The world is changing, and parasites adapt. The Nordic-Baltic region in northern Europe – including the Nordic countries Denmark, Finland, Iceland, Norway and Sweden, and the Baltic States Estonia, Latvia and Lithuania – is facing new parasitological challenges due to changes in populations of parasites and their hosts and the spread of new parasites to the region due to climate change. Some changes can also be ascribed to increased awareness and detection. In this paper, we review and discuss a convenience selection of ten timely examples of recent observations that exemplify trends and challenges from different fields of parasitology, with particular focus on climate change and potential changes in epidemiology of pathogens in northern Europe. The examples illustrate how addressing parasitological challenges often requires both intersectoral and international collaboration, and how using both historical baseline data and modern methodologies are needed.

## Graphical abstract

**Figure:**
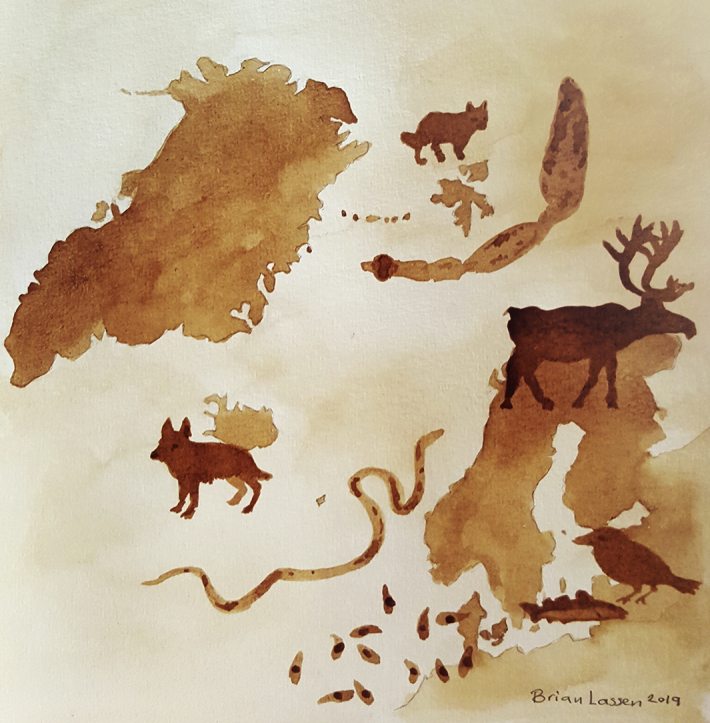

## 1 Introduction

The environmental change at high latitudes (Hoberg et al., 2017) is evident in the changing epidemiology of parasites in the Nordic-Baltic region in northern Europe. The region includes the Nordic countries Denmark, Finland, Iceland, Norway and Sweden, and the Baltic States Estonia, Latvia and Lithuania, and has a long history of collaborative studies within the field of parasitology. Changes in populations of parasites, their hosts, as well as their vectors have been observed, and new parasites have emerged in the region. Moreover, changes in management as well as movements and population dynamics of hosts affect the epidemiology of parasites in the region. In this paper, we review a selection of timely examples of recent observations.

## 2 Selection of examples

The Scandinavian-Baltic Society for Parasitology (SBSP, www.sbsp.eu) was established in 2003 by merging the Scandinavian Society for Parasitology (established in 1967) and the Baltic Society for Parasitology (established in 1993). After an invitation from the World Federation of Parasitologists to contribute to this special issue, the Board of the SBSP contacted the members of the society by email, asking for contributions to this work. All who expressed interest were welcomed to the working group. The examples represent a convenience selection.

### 2.1 *Trichinella* spp. infection in the sylvatic cycles: different patterns in Scandinavia vs. the Baltic States

*Trichinella* spp. remain highly endemic in the sylvatic cycles in some of the countries in the region, in particular the Baltic States and Finland (Bružinskaitė-Schmidhalter et al., 2012; Kirjušina et al., 2015; Deksne et al., 2016; Kärssin et al., 2017; Oksanen et al., 2018). There is a clear difference between the prevalence in sylvatic environment and domestic environment. For example, while 42.1% of free-ranging wild boars (*Sus scrofa*) hunted in Estonia tested positive for anti-*Trichinella* antibodies and Western blot -confirmed seroprevalence was estimated to be 17.4%, none of the tested domestic pigs were seropositive (Kärssin et al., 2016). This illustrates that biosecurity measures on domestic pig farms are efficient against *Trichinella* even in a highly endemic country. The number of reported human trichinellosis cases is relatively low in the region (EFSA and ECDC (European Food Safety Authority and European Centre for Disease Prevention and Control), 2018), but possible underdiagnosing or underreporting have not been evaluated.

In Estonia, prevalence of *Trichinella* spp. was 57.5% in raccoon dogs (*Nyctereutes procyonoides*), and 69.0% in red foxes (*Vulpes vulpes*) (Kärssin et al., 2017). *Trichinella nativa* was the most prevalent species in raccoon dogs and *T. britovi* in red foxes. In Latvia, *Trichinella* spp. larvae were detected in eight tested carnivore host species, with an overall prevalence of 49.2% (Deksne et al., 2016). *Trichinella britovi* is the most common species, followed by *T. nativa* – which is also regularly found in wild boars – while *T. spiralis* was detected in three animals as mixed infection with *T. britovi*. In Lithuania, *Trichinella* larvae were found in 46.6% of red foxes, and 29.3% of raccoon dogs (Bružinskaitė-Schmidhalter et al., 2012). In Finland, *Trichinella* larvae were detected in nine wild carnivore species out of ten tested, overall prevalence was 34.7% (Airas et al., 2010). All four European *Trichinella* species were detected, including *T. spiralis* which was found in lynxes (*Lynx lynx*), raccoon dogs, red foxes and one wolf (*Canis lupus*).

The biomass of circulating *Trichinella* spp. larvae has increased in Estonia and Latvia (Kirjušina et al., 2015; Kärssin et al., 2017). Interestingly, Scandinavia seems to have an opposite trend: for example in Norway, the infection prevalence estimates have decreased from above 20% to below 5% in red foxes, which was supported by investigation in one region with a decrease from over 5% to 1% (Davidson et al., 2006; Lundén, 2019) (unpublished observations, R.K. Davidson). In Finland, the overall *Trichinella* prevalence in wildlife has remained rather stable during the last few decades (Oksanen et al., 2018; Airas et al., 2010). However, the species composition of isolates has changed: disappearance of *T. spiralis* from the sylvatic cycle in Finland was explained by the eradication of infection from domestic pig farms, eliminating spillover to wildlife. In spite of climate change, the arctic species *T. nativa* appears so far not to have the slightest problem in thriving in the southernmost part of Finland and the Baltic States (Deksne et al., 2016; Kärssin et al., 2017; Oksanen et al., 2018).

### 2.2 Diversity seen when looked for: several new *Sarcocystis* species described during the last decade

Sarcocystosis is a neglected parasitic infection. During the last decade, increasing interest in *Sarcocystis* spp. in wildlife has led to description of 19 new *Sarcocystis* species in the region. This is an example of how understanding parasite diversity can only start after focusing and in-depth studies. It is noteworthy how the new species of *Sarcocystis* have been described in various intermediate hosts species: *S. arctica* and *S. lutrae* in Carnivora (Gjerde and Schulze, 2014; Gjerde and Josefsen, 2015; Kirillova et al., 2018; Prakas et al., 2018a); *S. albifronsi*, *S. anasi*, *S. corvusi*, *S. fulicae*, *S. halieti*, *S. lari*, *S. turdusi*, and *S. wobeseri* in birds (Kutkienė et al., 2010; Kutkienė et al., 2012; Prakas et al., 2014; Gjerde et al., 2018; Prakas et al., 2018b; Prakas et al., 2018c); *S. elongata*, *S. entzerothi*, *S. frondea*, *S. hjorti*, *S. nipponi*, *S. pilosa*, *S. silva*, and *S. truncata* in Cervidae (Dahlgren and Gjerde, 2010; Gjerde, 2012; Prakas et al., 2013; Prakas et al., 2016; Prakas et al., 2017; Rudaitytė-Lukošienė et al., 2018); and *S. ratti* in black rat (*Rattus rattus*) (Prakas et al., 2019).

Several data gaps have become evident. For most of the described species, the intermediate and definitive hosts remain unknown or presumed based on phylogenetic analyses. Among the described species both hosts are known for *S. halieti* (Gjerde et al., 2018; Prakas et al., 2018c) and *S. lari* (Prakas et al., 2014; Gjerde et al., 2018). According to available data these species are not of zoonotic significance.

Currently, two species *S. hominis* and *S. suihominis* are known as potential causative agents of human intestinal sarcocystosis (Poulsen and Stensvold, 2014). High prevalence of muscular sarcocystosis has been found in cattle, sheep, pigs and horses slaughtered for human consumption in Lithuania (Januskevicius et al., 2019). Previously, carnivores were assumed as definitive hosts of *Sarcocystis* spp., but recent studies have reported that carnivores act as intermediate hosts for *S. arctica* (Gjerde and Schulze, 2014; Kirillova et al., 2018) and *S. lutrae* (Gjerde and Josefsen, 2015; Kirillova et al., 2018; Prakas et al., 2018a). Additionally, investigation of invasive host species such as sika deer (*Cervus nippon*) have shown that it serves as an intermediate host of various species of *Sarcocystis*, including *S. entzerothi*, *S. frondea*, *S. nipponi* and *S. truncata* (Rudaitytė-Lukošienė et al., 2018) and *S. pilosa* (Prakas et al., 2016) detected in the Nordic-Baltic region. According to phylogenetic analysis, *Sarcocystis* sp. from sika deer found in Japan is related with *S. silva* and *S. truncata* detected in Lithuania (Abe et al., 2019); the latter has been reported as possible causative agent of food poisoning in Japan (Ota et al., 2019).

### 2.3 Anisakid parasites and crisis of the Baltic cod

The Baltic Sea, the largest brackish water area in the world, is located in the center of the Nordic-Baltic region, between Denmark, Sweden, Finland, Russia, Estonia, Latvia, Lithuania, Poland and Germany. Due to its low salinity, it is populated by a relatively low number of marine teleost species. The Baltic cod is a subpopulation of the Atlantic cod (*Gadus morhua*) which entered the region several thousand years ago. It is considered one of the most ecologically and economically important species in the area, but it is also vulnerable. The parasite-fauna of the Baltic cod has been studied extensively during the 20th century, and by the turn of the millennium major changes were observed concomitantly with occurrence of an unexpected crisis of the cod stock (Eero et al., 2015).

Nematode larvae of two anisakid species *Pseudoterranova decipiens* and *Contracaecum osculatum* were absent or infrequent findings in the Baltic cod in the 1980s and 1990s (Myjak et al., 1994; Haarder et al., 2014), but after the year 2000, high prevalence estimates and intensities were documented (Haarder et al., 2014; Perdiguero-Alonso et al., 2008; Buchmann and Kania, 2012; Mehrdana et al., 2014; Nadolna and Podolska, 2014; Rodjuk, 2014; Sokolova et al., 2018). The grey seal (*Halichoerus grypus*) is the final host of both species (Buchmann and Mehrdana, 2016; Zuo et al., 2018). From being a threatened species in the 1960s – comprising merely a few hundred individuals in the Baltic Sea – the grey seal population size is now estimated at a level between 30,000 and 40,000. Each individual seal can carry a high number of adult *C. osculatum* (Lunneryd et al., 2015) and each of these worms produce thousands of eggs daily which are delivered to the Baltic Sea with defecation. Investigations are being conducted to evaluate to what extent these parasites may explain the crisis of the Baltic cod; the historical records and data on host populations are important in this.

### 2.4 Changing reindeer parasite transmission patterns in the changing climate

The Fennoscandian reindeer (*Rangifer tarandus*) population numbers over 660,000 (Riseth et al., 2019) and includes semi-domesticated reindeer as well as wild tundra reindeer (*R. t. tarandus*) in the mountains of central Norway and forest reindeer (*R. t. fennicus*) in eastern and central Finland. These animals face increasing and interconnected anthropogenic and climatic challenges and changing infection pressures (Jokelainen et al., 2019; Tryland et al., 2019).

Elaphostrongylosis, caused by meningeal worm *Elaphostrongylus rangiferi*, is a snail-borne helminthiasis with temperature-dependent development in snails (Halvorsen et al., 1980; Josefsen and Handeland, 2014). Clinical signs include ataxia and hind-limb paresis that appear before larvae can be detected in the feces (Handeland and Slettbakk, 1994), and available treatment options are palliative. Long-term baseline data is patchy but sporadic cases are diagnosed across the region, except Iceland (Josefsen and Handeland, 2014; Skirnisson et al., 2006; Handeland et al., 2019). Warm summers in the early 1970s resulted in large elaphostrongylosis outbreaks in Finnmark (Halvorsen et al., 1980) with losses estimated at 22%. Similarly, during the exceptionally warm summer in 2018, reindeer herders from central Norway reported an outbreak of elaphostrongylosis. One herder reported losing 70 animals of all age groups (unpublished data, T. Mørk), of which three cases were confirmed. This outbreak was unusual not only for its timing – the disease is normally seen from early winter (Josefsen and Handeland, 2014) – but also for the wide range of ages that developed symptoms. The changing epidemiological and clinical picture is of considerable concern not only from an animal health and welfare perspective but also with regard to the sustainability and cultural aspects of traditional reindeer herding and the vulnerable last populations of wild tundra reindeer in central Norway.

Most probably linked to roe deer (*Capreolus capreolus*) invading northern Fennoscandia during the 1960s, their mosquito-transmitted filarioid nematode *Setaria tundra* now inhabits the reindeer husbandry area and causes outbreaks of peritonitis in reindeer calves in Finland (Laaksonen, 2010). The probability of an outbreak is high following two consecutive warm summers (June–August mean temperature exceeding 14 °C) enabling the development of the parasite in mosquito vectors (Laaksonen et al., 2010). The microclimatic preconditions to the development of *Setaria* larval infectivity have recently been further focused on (Haider et al., 2018).

The North American white-tailed deer (*Odocoileus virginianus*) was introduced into southern Finland in 1935. Obviously, it was accompanied with the lymphatic filarioid nematode *Rumenfilaria andersoni* which remained under the radar until the early 2000s, when it was found to be prevalent in Finnish reindeer, and later on, in all four cervid species in Finland (Laaksonen et al., 2010). While it can be assumed to spread further, its vector has not been identified yet in spite of investigating thousands of potential vectors.

### 2.5 Transmission of bird malaria in the north

Blood parasites causing malaria and other haemosporidioses in birds are widespread all over the world (Valkiūnas, 2004). Pathogens belonging to *Plasmodium*, *Haemoproteus* and *Leucocytozoon* genera are transmitted by vectors, mainly Culicidae, Ceratopagonidae and Simuliidae, respectively (Santiago-Alarcon et al., 2012). Haemosporidians can cause outbreaks in farms, zoos, aviaries and wild bird populations (Valkiūnas, 2004). Some avian malarial parasites, including *Plasmodium relictum* that is listed in top ten of the most invasive species in the world (Lowe et al., 2000), are already transmitted in northern Europe.

More than 60 morphological species of *Plasmodium* have been described and many more genetic lineages recorded (Bensch et al., 2009; Valkiūnas and Iezhova, 2018). Recent studies showed that some of them are already prevalent and transmitted by mosquitoes up to the northern arctic circle (Marzal et al., 2011; Loiseau et al., 2012), while others are found only in long-distance migrant birds after annual migration to Africa. Wild birds serve as reservoirs for outbreaks of new diseases. Populations of birds in the north, including the Nordic-Baltic region, which do not migrate, or migrate only within Europe, may be at risk of having contacts with new pathogens. A model based on empirical data collected from Arctic region has demonstrated that local birds may be exposed to new species of malarial parasites in near future under climate warming conditions (Loiseau et al., 2012). Evaluation of avian malaria prevalence together with air temperatures in last seven decades revealed that increase of a global temperature by 1 °C co-occurs with two- to threefold increase of avian malaria prevalence (Garamszegi, 2011). After comparing all continents the biggest effect was found in Europe and Africa. These predictions alarm about significant changes of epidemiological situation in near future.

### 2.6 Canine vector-borne parasites are imported and spreading

Canine vector-borne parasites are emerging, spreading and of increasing clinical relevance in domestic dogs in the Nordic-Baltic region. By importing dogs and traveling with dogs, humans facilitate this change, which is a challenge for practicing veterinarians (Tiškina and Jokelainen, 2017; Mikola et al., 2020).

Import of dogs to Iceland is restricted (Skirnisson et al., 2018). The current Icelandic fauna of canine parasites is not known to include vector-borne parasites (Skirnisson et al., 2018). Some parasites have been eradicated through successful campaigns, some by elimination of the vector or intermediate host (Skírnisson, 2017). For decades, lack of blood sucking vectors in the country set the focus on looking only for intestinal parasites in fecal samples from the imported animals. In the recent years the population of biting midges (*Culicoides reconditus*) has been on the rise, illustrating the changes in the vector situation and reminding of the possibility of parasite life cycles including different vectors. The situation may change quicker than policies adapt.

The zoonotic parasite *Dirofilaria repens* was previously reported as sporadic travel-related finding in humans and dogs in the Nordic-Baltic region (Tiškina and Jokelainen, 2017; Sævik et al., 2014; Klintebjerg et al., 2015; Vatne, 2015; Capelli et al., 2018). Now it has established its life cycle in the Baltic States (Melbarde-Gorkusa et al., 2011; Stepanjana et al., 2012; Jokelainen et al., 2016; Sabūnas et al., 2019), and the northernmost autochthonous human infection has been described from Finland (Pietikäinen et al., 2017). The local presence of the parasite is not yet widely known to the medical profession (Mikola et al., 2020).

*Angiostrongylus vasorum* is common in Denmark (Saeed et al., 2006; Taubert et al., 2009; Al-Sabi et al., 2013; Al-Sabi et al., 2014) and established in Sweden (Åblad et al., 2013; Grandi et al., 2017) and Finland (Isomursu et al., 2010; Tiškina et al., 2019). In Norway, *A. vasorum* was detected in a red fox for the first time in 2016 (Norwegian Veterinary Institute, 2018). In Estonia, *A. vasorum* has been described in red foxes and raccoon dogs (Laurimaa et al., 2016a; Laurimaa et al., 2016b), but not in domestic dogs. In Finland, it was described in red foxes several decades before the first autochthonous cases in domestic dogs were described (Tiškina et al., 2019).

Other canine vector-borne parasites that are currently relevant include *Babesia canis*, which is changing from a mainly imported parasite to locally present parasite in many areas in the Nordic-Baltic region (Tiškina and Jokelainen, 2017; Øines et al., 2010; Berzina et al., 2013; Paulauskas et al., 2014; Stensvold et al., 2015; Tiškina et al., 2016; Capligina et al., 2016; Radzijevskaja et al., 2018). *Leishmania* has a similar history: first imported canine case to Denmark was reported in international literature in 1985 (Bindseil et al., 1985), and now local non-vector transmission has been described in Finland (Karkamo et al., 2014).

### 2.7 *Echinococcus multilocularis* – well-established in the Baltic States, emerging in Scandinavia, while Finland and mainland Norway considered free

*Echinococcus multilocularis* is endemic in the Baltic States, and alveolar echinococcosis causes a worrying disease burden e.g. in Lithuania (Laurimaa et al., 2016a; Marcinkutė et al., 2015; Bagrade et al., 2016). In the Nordic countries, this zoonotic parasite was first reported in Denmark in 2000 near Copenhagen (Saeed et al., 2006), and on the island of Svalbard, Norway, in 2001 (Henttonen et al., 2001). In Svalbard the parasite was likely introduced either with the introduction of the parasite's intermediate host, the sibling vole (*Microtus levis*, also known as *Microtus rossiaemeridionalis*) or through migration of infected arctic foxes (*Vulpes lagopus*) on pack ice from the mainland (Henttonen et al., 2001; Knapp et al., 2012).

Follow-up surveys have revealed other hot spots in Denmark (Enemark et al., 2013; Wahlström et al., 2015; Petersen et al., 2018). In 2011 the parasite was identified in Sweden (Osterman Lind et al., 2011), and by 2014 several hot-spots were found (Wahlström et al., 2015; Miller et al., 2016a; Miller et al., 2016b). Recent genetic work using the DNA microsatellite marker EmsB suggests that migrating infected foxes or domestic dogs could have introduced the parasite in both Denmark and Sweden (Knapp et al., 2019). The overall prevalence in red foxes in Sweden is ≤1% (Wahlström et al., 2015), but the proportion of fox feces with eggs has been >50% in localized areas (Miller et al., 2016b). The absence of key intermediate rodent hosts, namely the common vole (*Microtus arvalis*) and a specific water vole (*Arvicola scherman*), could be a limiting factor for the parasite in Sweden (Miller, 2016, Miller et al., 2016a; Knapp et al., 2019). Autochthonous human alveolar echinococcosis cases have not been reported from Denmark. In 2019, the Swedish Institute for Infectious Disease Control stated that autochthonous infection cannot be ruled out for three recent human cases diagnosed in Sweden (Swedish Institute for Infectious Disease Control, 2019).

Mainland Norway and Finland are considered *E. multilocularis* free (Wahlström et al., 2015; Davidson et al., 2016), and there is an EU directive on preventive health measures for entering dogs (Commission Delegated Regulation (EU), n.d.). This is a unique legislative action addressing domestic dogs to protect human health from a zoonotic parasitic disease.

### 2.8 Parasite-friendly eating trends

Lately, zoonotic foodborne parasites have gained attention at global and European level (FAO/WHO, 2014; Bouwknegt et al., 2018; EFSA Panel on Biological Hazards (BIOHAZ) et al., 2018). A multicriteria ranking exercise identified *E. multilocularis*, *Toxoplasma gondii* and *Trichinella spiralis* as the most important parasites for Europe (Bouwknegt et al., 2018).

The concern that forest berries could carry *Echinococcus* infections to humans (Kern et al., 2004) is relevant for the Nordic-Baltic region, where collecting forest berries is popular. Recent experimental studies using non-zoonotic *Taenia laticollis* eggs as a model showed that forest berries can serve as a vehicle for taeniid eggs (Malkamäki et al., 2019). However, infection from ingestion of berries contaminated with parasite eggs has yet to be proven conclusively (Lass et al., 2015; Robertson et al., 2016; Lass et al., 2016). Recently, a study on individuals with cystic echinococcosis compared with matched controls did not identify picking wild berries or mushrooms to be among the locally relevant risk factors (Laivacuma et al., 2019).

*Toxoplasma gondii* is common in animals raised and hunted for human consumption in the region (Olsen et al., 2019) as well as in the main definitive hosts, domestic cats (Jokelainen et al., 2012; Deksne et al., 2013; Must et al., 2015; Must et al., 2017). The wild felid living in some parts of the region, Eurasian lynx, has not been shown to shed oocysts of *T. gondii* (Jokelainen et al., 2013; Ryser-Degiorgis et al., 2006). Genotype II is the most common *T. gondii* genotype detected from animals in the region (Jokelainen et al., 2012; Prestrud et al., 2008; Jokelainen et al., 2011; Jokelainen and Nylund, 2012; Jokelainen and Vikøren, 2014). Because genotyping data are not available from animal hosts from most countries in the Nordic-Baltic region, it can only be speculated whether imported foodstuffs and international mobility partly explain the recently reported wider genetic diversity of *T. gondii* in human clinical samples from Denmark (Jokelainen et al., 2018).

*Trichinella* spp. remain important zoonotic foodborne parasites in the region. Here, the recent finding of *T. britovi* in European beaver (*Castor fiber*) (Segliņa et al., 2015) merits a mention, because there is increased interest among hunters to use beaver meat for consumption. The European beaver is an herbivore and thus not a classical host for *Trichinella* nematodes, that are transmitted by carnivorism and scavenging.

### 2.9 Outbreaks of cryptosporidiosis and giardiasis – why so many in the Nordic countries vs. Baltic States?

In 2013, a publication addressed the question of whether foodborne cryptosporidiosis really did occur more often in Nordic countries than elsewhere, as suggested by a skewed distribution of reporting (Robertson and Chalmers, 2013). After examining various aspects, the authors concluded that the next outbreak of foodborne cryptosporidiosis was no more likely to occur in the Nordic countries than elsewhere. Since the outbreaks summarized in that publication, a further seven foodborne outbreaks of human cryptosporidiosis have been recorded in the international literature, of which four are from Scandinavia – one from Finland, one from Norway, and two from Sweden (EFSA Panel on Biological Hazards (BIOHAZ) et al., 2018; Robertson et al., 2019).

In addition, within recent decades some notable waterborne outbreaks of cryptosporidiosis and giardiasis have been recorded from Nordic countries, with around 6000 cases of giardiasis reported from a waterborne outbreak in Norway in 2004 and around 47,000 cases of cryptosporidiosis reported from two outbreaks in Sweden in 2010 and 2011 (Guzman-Herrador et al., 2015). In contrast, there are no records of food- or waterborne outbreaks of human cryptosporidiosis or giardiasis from the Baltic States. This may reflect that much of the drinking water supply there is ground water, whereas in Sweden and Norway surface waters are commonly used as drinking water sources and these are also more vulnerable to contamination. However, the lack of foodborne outbreaks reported to date from the Baltic States probably reflects that in these countries fewer resources are directed towards investigation and reporting of small outbreaks than in Nordic countries. However, with the recent greater emphasis on the presence of these parasites and their transmission in Baltic countries (Santoro et al., 2019; Plutzer et al., 2018), it might be expected that future outbreaks can be expected from these countries also, and that public health officials should be prepared for these events.

### 2.10 Common human intestinal parasites cause clinical dilemmas

The improvements in diagnostic methods available have resulted in an increase in number of findings of *Dientamoeba fragilis*, an intestinal protozoan with debated clinical significance. In Finland, it is the most common parasite detected in the capital region (Pietilä et al., 2019). In Denmark, *D. fragilis* is common in the apparently healthy adult population (Krogsgaard et al., 2015), and commonly detected in stool samples from children that have been taken for diagnostic purposes (Röser et al., 2013). In Sweden, one study reported that gastrointestinal symptoms coincided with prevalence of finding *D. fragilis* (Ögren et al., 2015). A study on children in Danish daycare showed high prevalence, reaching 100% during the course of the cohort study, and found no difference in prevalence in reportedly asymptomatic children and in children who reportedly had had gastrointestinal symptoms (Jokelainen et al., 2017). The study also evaluated whether high parasite load, evaluated by real-time PCR, was associated with presence of symptoms, and there was no statistical association with recent gastrointestinal symptoms, only with reported weight loss.

*Dientamoeba fragilis* is an example of how new diagnostic methods may put medical doctors in challenging situation: to test or not, to treat or not. As the conclusions about the clinical importance of this parasite seem to differ in different countries around the world, international collaborative studies are called for.

## 3 Discussion

The Nordic-Baltic region in northern Europe is facing several parasitological challenges due to changes in populations of parasites and their hosts and the spread of new parasites to the region due to climate change. Some changes can also be ascribed to increased awareness and detection. While some of the trends differ by country within the region, many of the challenges are common to the whole northern part of the world (Hoberg et al., 2017; Jenkins et al., 2013), emphasizing the need for comparative regional studies and wider international collaborations.

The wildlife of the region hosts numerous parasites, including many that can also infect domestic animals and humans. The risk of spillover to and from domestic animals needs to be addressed continuously by biosecurity practices and monitoring. Because wildlife is hunted for human consumption, studies yielding data for source attribution of tissue-dwelling foodborne parasites should include not only domestic animals, but also game (Olsen et al., 2019). Parasitological challenges involving wildlife often require intersectoral collaboration and must take into account the changes in host populations and environmental parameters.

In the Nordic-Baltic region, the red fox and raccoon dog populations have been affected by other factors, such as rabies eradication and scabies (Veeroja and Männil, 2015; Robardet et al., 2016), and currently African Swine Fever and its management are affecting the wild boar and pig populations in the Baltic States (Sanchez-Vizcaino et al., 2015; Schulz et al., 2019). Longitudinal and repeated studies are needed to monitor how these changes affect the epidemiology of e.g. *Trichinella* spp., *Echinococcus* spp. and *T. gondii*. The reasons for the opposite trends in *Trichinella* prevalence in red foxes observed in the Baltic States vs. Scandinavia would be important to study, and the role of new host species establishing themselves in the region needs to be taken into account.

Selecting suitable methodologies is also important: in many cases traditional methods may have underestimated the diversity, and it seems that the modern molecular methods could easily replace them. However, caution is needed when changing to modern methodologies. For example, recent studies have revealed that PCR-based detection of avian malaria can underestimate the commonly occurring multiple infections with closely related parasites (Pérez-Tris and Bensch, 2005; Valkiūnas et al., 2006; Zehtindjiev et al., 2012) and fail to provide information about the host range (Valkiūnas et al., 2014a; Moens et al., 2016). The first issue was documented by comparative studies using microscopy and PCR from wild caught birds infected with haemosporidian parasites and experimental study after amplification of haemosporidian parasites DNA mixes (Valkiūnas et al., 2008; Bernotienė et al., 2016): it was revealed that general primers designed for amplification of closely related parasite's DNA are selective during mixed infections. The second issue was detected when DNA of parasite was amplified from host blood sample, but infective stages crucial for transmission of pathogen were not found using microscopy, demonstrating that presence of parasite's DNA does not mean that the vertebrate or invertebrate species is a real host of the parasite (Valkiūnas et al., 2014a; Moens et al., 2016; Valkiūnas et al., 2009). Both correct estimation of parasite composition in a single host and determination of host range are crucial for understanding the epidemiology host-parasite interactions, and drivers for emergence and spread of infectious diseases. Thus, at present, in many cases it is prudent to use both molecular and microscopic examination in parallel (Valkiūnas et al., 2014b), especially when dealing with organisms which are evaluated as potential hosts for the first time.

Regardless of detection methods, one has to first look for the parasites to find them. It is a typical story that when a parasite is gaining attention, it becomes evident that it has been there longer, only unnoticed, or that the diversity is actually greatly wider than previously assumed. Awareness is the key to detect emerging parasites. Challenge may also be to ensure awareness in all related fields, for example for the emerging zoonotic vector-borne parasites of dogs (Mikola et al., 2020). Strengthening intersectoral and international collaboration for research and monitoring would help in addressing and enable efficient science-to-policy actions in all relevant fields of emerging parasitological One Health challenges (Aguirre et al., 2019).

The vector forecast of the Nordic-Baltic region indicates that awareness is needed. Climate change has direct and indirect influence on successful development and prevalence of several vector-borne parasites. For avian malaria pathogens, for example, environmental temperature directly affects the development in insects (Garamszegi, 2011; Paaijmans et al., 2009), and due to global warming the timing of migrant arrival and overlapping habitats with vectors can result in new contacts with pathogens (Fuller et al., 2012). Moreover, invasion of new vectors will further affect the situation. For parts of the Nordic-Baltic region, it is also relevant that the spread of vector-borne infectious diseases goes not only horizontally towards north, but also vertically up to the mountains (Danielová et al., 2010).

Climate change will, however, not only affect vector-borne parasites. For example, warmer temperatures may enable range expansion of different host species and the parasites they carry with them, winter freezing may be less likely to inactivate environmental transmission stages, and extreme weather events are likely to have impact on water contamination, and thereby the dissemination of waterborne, and some foodborne, parasites. In addition, globalization of both the food trade and of people may result in the introduction of parasites that are believed not to be present in some countries in the Nordic-Baltic region or to have been eliminated (e.g., *Taenia solium*; Laranjo-González et al., 2017; Trevisan et al., 2018), or the introduction of potentially more virulent strains of parasites that are endemic (e.g., more virulent *T. gondii* strains; Jokelainen et al., 2018). The interconnectedness between changes and outcomes is complicated and difficult to predict, and supports that such challenges should be tackled from a One Health perspective.

The examples covered in this review also illustrate outcomes from research collaborations in the region. The combination of carefully recorded baseline data from the long history in parasitology with modern approaches and international collaborations are a strength in maintaining and strengthening preparedness for future challenges in our changing world.
